# Amniotic membrane application in surgical treatment of conjunctival tumors

**DOI:** 10.1038/s41598-023-30050-y

**Published:** 2023-02-17

**Authors:** Alena Furdova, Gabriela Czanner, Jan Koller, Pavol Vesely, Robert Furda, Zuzana Pridavkova

**Affiliations:** 1 Department of Ophthalmology, Faculty of Medicine, Comenius University, Bratislava, Slovakia; 2 Liverpool John Moores University, Liverpool, UK; 3 Faculty of Informatics and Information Technology, Slovak University of Technology, Bratislava, Slovakia; 4 Department of Burns and Reconstructive Surgery, Faculty of Medicine, Comenius University, Bratislava, Slovakia; 5 VESELY Eye Clinic, Bratislava, Slovakia; 6 Department of Information Systems, Faculty of Management, Comenius University, Bratislava, Slovakia; 7 UVEA Mediklinik, Martin, Slovakia

**Keywords:** Eye cancer, Surgical oncology

## Abstract

The amniotic membrane (AM) has special properties, making it ideal for clinical applications in various surgical fields like ophthalmology. It is used more frequently to cover conjunctival and corneal defects. In our retrospective study we have been combined 68 patients with epibulbar conjunctival tumors they have been surgically treated in the period of 2011–2021. Seven (10.3%) patients have been treated with AM application after surgical removal of the tumor. 54 (79%) cases were malignant, and 14 (21%) were benign. In the analyzed dataset the males had just slightly higher chance of malignancy than females, 80% versus 78.3%. For the significancy calculation the Fisher exact test was used and the result proved no significancy (*p* = 0.99). Six patients with AM application were malignant. The observed difference in the number of quadrants of the bulbar conjunctiva infiltrated versus significant malignancy with *p* = 0.050 calculated by Fisher Exact test and with *p* = 0.023 calculated by Likelihood-ratio test. The results of our study indicate that AM grafts are an effective alternative to cover defects after removal of epibulbar lesions due to their anti-inflammatory properties because the conjunctiva must be preserved, and especially the most important application is in malignant epibulbar conjunctival tumors.

## Introduction

The amniotic membrane (AM) is a thin, translucent biological structure with no nerves, blood, or lymph vessels. Its innermost layer of the placenta is located right next to the foetus with a thickness that varies from 0.02 to 0.5 mm. It consists of three fundamental histological layers: epithelial layer, basement membrane and avascular mesenchymal tissue^1^. The inner layer consists of a single homogenous layer of cuboidal epithelial cells, firmly attached to the basement membrane. These cells possess numerous microvilli on their apical surface and it`s thought to participate in intra- and extracellular transport functions along with secretory function^2^. The basement membrane is composed of heparan sulfate rich proteoglycans enabling it to act as a permeable barrier to amniotic macromolecules and structural molecules maintaining the membrane integrity^3^. The outer layer, the avascular stroma, contains mostly mesenchymal stromal cells and collagen I, III, IV, and VI that increase its tensile strength^4^. The AM has special properties, making it ideal for clinical applications in various surgical fields such as urology, ophthalmology, oncology and plastic surgery^5^.

The most important feature of AM is lack of immunogenicity, both cell types contain only limited HLA class Ia antigens^6^. Implantation of cryopreserved AM observed no immunological rejection signifying no need for immunosuppressive treatment after AM transplantation^7^.

The AM produces various numbers of growth factors, cytokines and vasoactive peptides, which allow for epithelialization and support cell proliferation. Expression of growth factors is preserved even at − 80 °C^8^. AM has anti-inflammatory, anti-fibrotic, anti-angiogenic, and anti-microbial properties documented in many studies making it attractive option in treatment plan^9–12^.

The first ever use of AM as a biological dressing was by Davis in 1910 for skin transplantation^13^. In ophthalmology, it was attempted to use a fetal membrane to reconstruct an ocular surface in patients with symblepharon by de Rötth^14^. In 1965, a procedure for sterilizing AM was developed, allowing it`s use on acute second-degree burns^15^. Kim and Tseng, in 1995, published a work that allowed the modern use of AM in ophthalmology to this day^16,17^. AM can be used at most 14 days after harvesting when stored at + 4 °C, but because donor testing must be finished first, it`s unsuitable to be used immediately^5^.

There, it is more profitable to preserve AM using one of the three prevalent methods: cryopreservation, using dimethyl sulfoxide (DMSO) as cryoprotectant^18^, glycerolization, eliminating all pathogens and 85% glycerol at + 4 °C, was shown to obliterate the HIV-1 virus^19^, and lyophilization^20^. At the Bratislava Central Tissue Bank (CTB) was developed method for cryopreserving AM using polyester net, membranes preserved with this method has shown outstanding outcomes when used in ophthalmology, burn medicine, or plastic surgery^5^. Cryopreservation of AM better preserves basement membrane components so it`s more suitable for cell cultivation, because they release more soluble wound-healing-modulating factors compared to other methods of preservation^21^. AM preparation is a complex task, everything starts with a fetal membrane procured according to CTB`s Standard Operating Procedure. After cesarean section, fetal membrane is removed from the placenta and transported in a sterile container to the CTB, where it`s aseptically processed. Processing started by rinsing in sterile 0,9% saline solution and then incubation overnight at + 4 °C in antibiotic cocktail (100 µg/ml ceftazidime and 25 µg/ml amphotericin B). When the incubation period is over, AM is separated from the chorion by blunt dissection, cleaned from blood remnants and stretched on a sterile polyester net with the epithelial surface facing up. Depending on the further intended use, the membrane along with a polyester net is cut up to different sizes and edges of such prepared grafts are fixed to the carrier by silver neurological clips. These amniotic sheets are then rinsed in cryoprotective freezing medium, sealed in sterile cryobags and placed in quarantine deep freezer at − 70 °C awaiting approval for distribution and clinical use, after which they are transferred to storage freezers where they can be stored up to 5 years^5^. Before surgery it`s needed warm AM at room temperature, the entire package is immersed in excess room temperature water and briefly lavaged until the tissue is thawed. After removal from the water bath and drying of the package, the outer cover bag is opened. Subsequently, the PA package is opened under aseptic conditions and the transplant is transferred with sterile forceps to an excess of lukewarm sterile saline solution to remove the cryoprotective medium. Every package with AM is provided with a unique identification tissue label containing the transplant code, tissue dimensions, date of preparation and date of expiration. Thawed AM graft must be used within 24 h. In ophthalmology is AM used mainly to promote epithelization, to reduce pain and to minimize the inflammation of the ocular surface. The integration into host tissue associated with the formation of desmosomes and hemidesmosomes provides stability for regenerating epithelium^22,23^.

AM grafts are predominantly used in indications of epibulbar lesion surgical removal with covering the defect e.g., corneal ulcers, corneal perforations, pterygium, bullous keratopathy and conjunctival reconstruction after epibulbar conjunctival tumor surgery^24^.

In our study, we analyzed the application of an AM graft after excision of epibulbar conjunctival tumor. Our aim was to emphasize the constant importance of using AM in ophthalmology, to explore the association between the malignancy and the number of quadrants covered in lesions, as well as the association between the malignancy and the need to apply AM. The patients in our study have been proven by epibulbar tumors that form a very heterogeneous group in terms of the clinical picture and histological structure. We did not investigate the proportion of malignant tumors occurring in men or women, describe any results concerning scarring and tumor recurrence, or compare whether the AM was applied more frequently in malignant or benign tumors. In the differential diagnosis of these tumors, it is necessary to distinguish between inflammatory and post-inflammatory changes, or between dystrophic and degenerative changes of the conjunctiva. The etiology of epibulbar tumors is in most cases multifactorial (age, overexposure to ultraviolet radiation, presence of pre-existing conjunctival pigment lesions, HIV—human immunodeficiency virus, HPV—human papilloma virus). Conjunctival tumors are divided into two groups based on the presence or absence of melanocytes. Non-pigmented (non-melanocyte) tumors are predominantly benign, whereas pigmented (melanocyte) tumors often undergo malignant transformation or are primarily malignant. Tumors arising by spread per continuitatem, most commonly in intraocular melanoma, are referred to as secondary. The AM topic is quite broad, therefore, in the discussion we compare not only our results but also we describe several aspects that relate to the AM in ophthalmology.

## Material and methods

### Study design, setting and participants

We have conducted a retrospective study of patients with epibulbar conjunctival tumor. We extracted data on 68 consecutive patients with conjunctival growth, be it malignant or benign in nature within time frame of 2011–2021. 54 (79%) cases were malignant, and 14 (21%) were benign.

Surgery was performed under topical or local anesthesia. In patients with melanoma or carcinoma verified by frozen section biopsy, we also applied mitomycin C after surgical tumor removal. Histopathology standard hematoxylin–eosin was performed on every patient. AM was sutured by resorbable sutures. Patients after surgery were regularly observed after healing at 3-, 6-, and 12-months intervals.

### Variables, data sources and management

We monitored whether the lesion of the conjunctiva outgrowth cornea and how many quadrants had been infiltrated. Then, we observed how each patient was treated and if AM was applied.

### Bias, study size, quantitative variables

There was no formal sample size calculation done, due insufficient previous published relevant work. The study size was determined by consecutive extraction of all eligible patients’ data. If any data are missing, the pattern of missingness is investigated to assess the potential for bias due to missing data. The only quantitative variable is age, which we do not put into groups.

### Statistical methods

To study the association between two categorical variables (e.g., malignancy and gender) we used Fisher exact test and Likelihood-ratio test, whenever the expected counts in a table cell were less than 5, otherwise we used a Pearson Chi-square test which is an approximation. Statistical analysis was conducted with SPSS software. For all analyses we use level of significance of 0.05. We do not adjust for multiple comparisons, as this is an exploratory study.

### Ethics and inclusion statement

All methods were performed in accordance with the relevant guidelines and regulations. This study complies with the guidelines for human studies and animal welfare regulations, the guidelines of the Helsinki Declaration have been adhered to. The treatment of all subjects that was described in this study was performed after gaining written informed consent, which was obtained from all patients and/or their legal guardians and is available in the patients’ records according to the local legislation and regulations. The documented confirmation for the agreement for publishing contains that the experimental protocol was approved by the ethics committee “Ethics Committee University Teaching Hospital Bratislava” by including the statement No. EC/139/2022.

## Results

**Figure 1:**
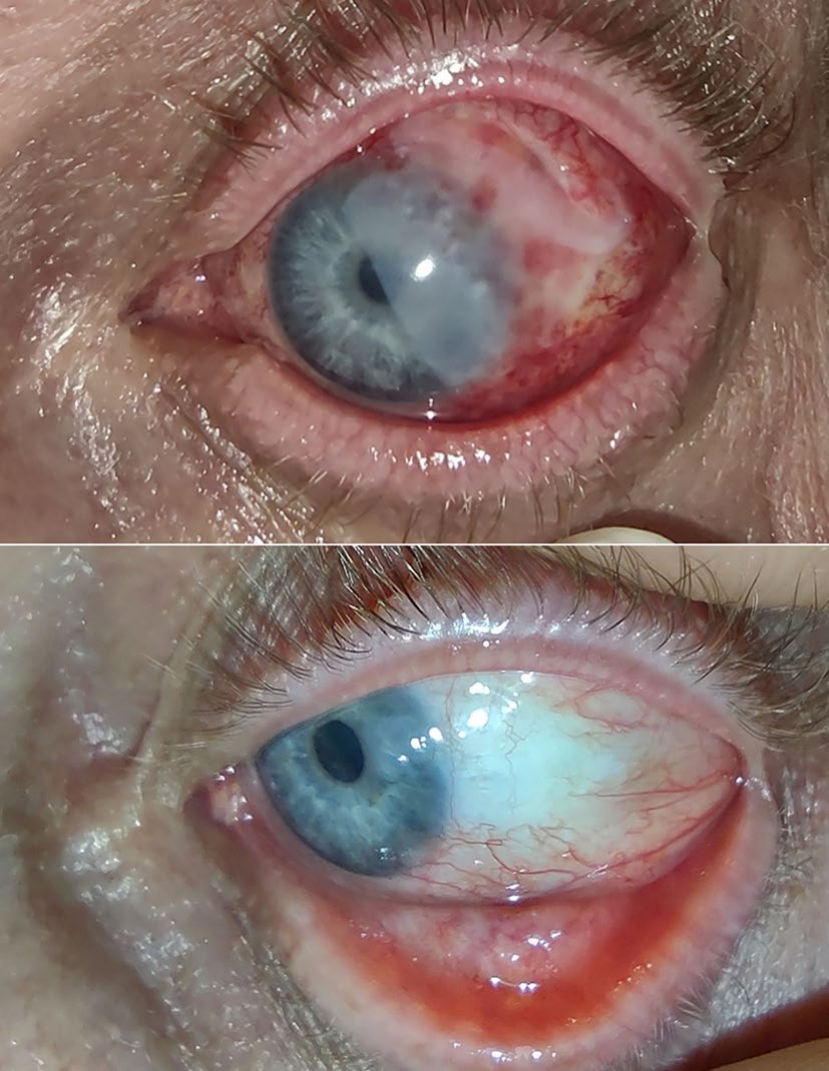
The anterior segment picture of patients 2 days (upper) and one year (lower) after epibulbar carcinoma surgery with the application of AM.

**Figure 2:**
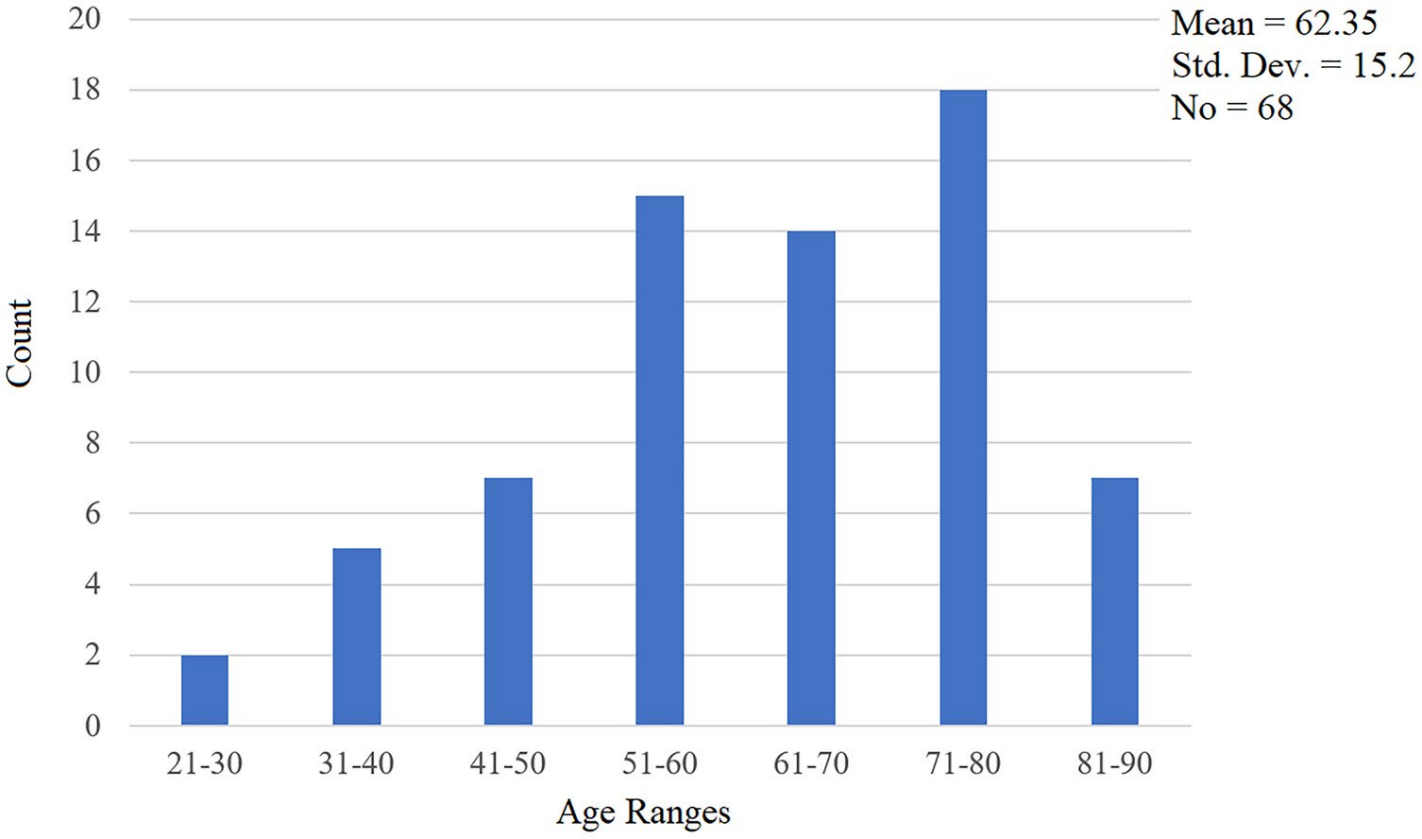
Age groups in 68 patients in decades.

In this retrospective study we analyzed within 2011–2021 timeframe 68 patients with epibulbar conjunctival tumors. Seven (10.3%) patients have been treated with AM application after surgical removal of the tumor. and the cosmetic results after surgery with AM application were good (Fig. 1). The age of the patients was 25 to 86 years. For better visualization the age has been grouped in decades (Fig. 2), what showed the significant representation of the patients between fifty and eighty years.

From the gender perspective, there have been 45 (63.2%) males and 23 (36.8%) females (Table 1). The malignant tumor was confirmed in 54 (79.4%) cases, and 14 (20.6%) cases were benign. In our dataset the males had just slightly higher chance of malignancy than females, 80% versus 78.3%. The observed difference was not significant with the *p* = 0.99 calculated by Fisher exact test and the *p* = 0.867 calculated by Likelihood-ratio test. Therefore, our result cannot be generalized.

**Table 1:** A group of 68 patients/gender versus malignancy.

Gender	Malignancy		Total
	No	Yes	
Male	9 (20%)	36 (80%)	45 (100%)
Female	5 (21.7%)	18 (78.3%)	23 (100%)
Total	14 (20.6%)	54 (79.4%)	68 (100%)

In the second analysis, we investigated the association between the number of quadrants infiltrated and malignancy of the lesion (Table 2). In patients with one quadrant infiltrated, there were 22 (66.7%) malignant cases. In patients with two quadrants infiltrated, there were 25 (89.3%) malignant cases. In patients with three or four quadrants infiltrated, there were 7 (100%) malignant cases. All cases with 3 or 4 quadrants were confirmed the malignancy. Hence, there was a clear increasing trend in our sample, and this trend was found to be statistically significant (*p* = 0.016, Fisher Exact test and Chi-squared test). Additionally, the observed difference was significant with the *p* = 0.050 calculated by Fisher Exact test and with the *p* = 0.023 calculated by Likelihood-ratio test. Therefore, these results can be generalized.

**Table 2:** A group of 68 patients/number of infiltrated quadrants versus malignancy.

Number of quadrants	Malignancy		Total
	No	Yes	
1	11 (33.3%)	22 (66.7%)	33 (100%)
2	3 (10.7%)	25 (89.3%)	28 (100%)
3 or 4	0 (0.0%)	7 (100%)	7 (100%)
Total	14 (20.6%)	54 (79.4%)	68 (100%)

In the third analysis, we investigated the association between cases with AM application and the malignancy (Table 3). Among patients with malignancy, there were 6 (11.1%) cases with AM application. Among patients without malignancy, there was only 1 (7.7%) case with AM application. Hence, patients with malignancy were more likely to require AM application, but this increase (4%) was not significant with the *p* = 0.98 calculated by Fisher exact test and the *p* = 0.651 calculated by Likelihood-ratio test. Therefore, our results cannot be generalized.

**Table 3:** A group of 68 patients/AM application versus malignancy.

AM application	Malignancy		Total
	No	Yes	
No	13 (21.3%)	48 (88.9%)	61 (100%)
Yes	1 (14.3%)	6 (85.7%)	7 (100%)
Total	14 (20.6%)	54 (89.4%)	68 (100%)

**Figure 3:**
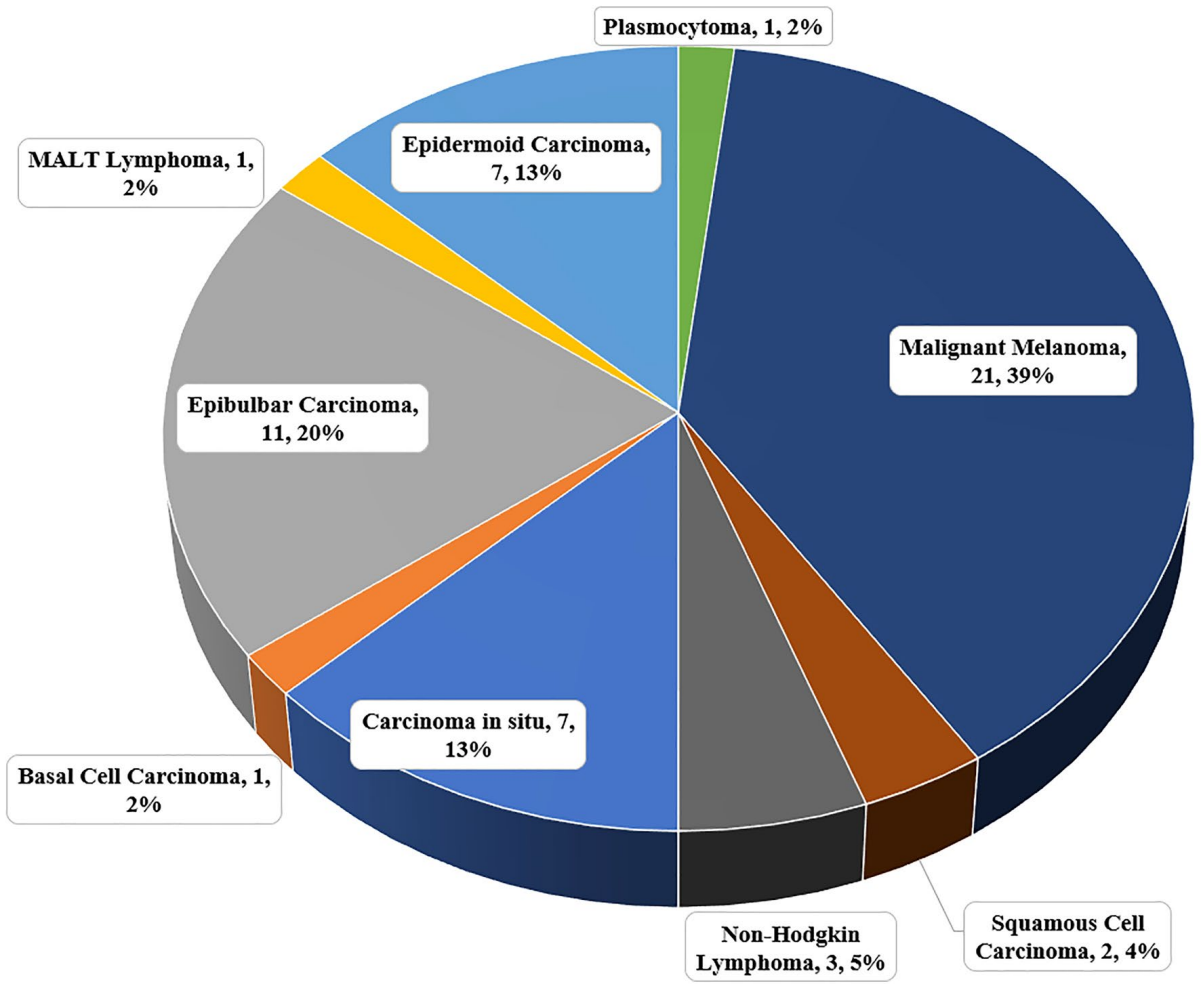
Histopathological findings in 57 patients with epibulbar malignancies.

In our study, the frequency of malignant tumors was the following: Carcinoma in situ 7 cases, Basal Cell Carcinoma 1 case, Epibulbar Carcinoma 11 cases, MALT Lymphoma 1 case, Epidermoid Carcinoma 7 cases, Plasmocytoma 1 case, Malignant Melanoma 21 cases, Squamous Cell Carcinoma 2 cases, and Non-Hodgkin Lymphoma 3 cases (Fig. 3).

## Discussion

Our experience is that after excision of the tumor with clear margins, or epibulbar lesion, the aim was to get the optimal functional, but also cosmetic results. To cover defects after excision, the suitable ‘filler’ should not induce scarring inflammation or vascularization. The healing of the grafted tissue, that should be necrosis-resistant or ischemic changes, needs to remain transparent, and the aim is to avoid the recurrence. During the surgery, manipulation with AM is easy and it can help the native cells to grow, for example, epithelial cells, goblet cells, which helps to migrate the epithelial conjunctival cells. On the epibulbar surface the goblet cells allow wetting and prevent the development of dry spots, help in the healing process, and AM downregulates inflammation and fibrosis^11^.

The allogenic graft should have ideally anti-cancer properties and should be non-immunogenic. AM helps in the reconstruction mainly in the large defects of the conjunctiva after large epibulbar lesions excision^25^.

Every surgery needs the “clear surgical margins” but also the epibulbar lesion removal. After excision, it often remains the large epibulbar defects they may require reconstruction. Large defects, more than 1 cm in diameter, can result in unsatisfactory direct closure, and the result may be the scarring or the restriction of eye movements, or a foreign body feeling that led to discomfort for the patient^26,27^.

De Roth was the first to introduce amnion to conjunctival reconstructive surgery in 1940^14^, however, the concept of AM use did not spread into ophthalmology until the 90 s, when Tseng et colleagues described the use of AM^28,29^. Their first step was to use the AM to treat the pterygium, corneal epithelial defect, symblepharon, and neoplasia that along with anti-angiogenic properties to prevent chronic inflammation, granulation tissue formation, and scarring^30^.

Several reports informed about the success of using the AM in the surgery for reconstruction defects after conjunctival tumor excisions^31–33^. Shields et al.^32^ first informed about the use of AM in the patient with epibulbar melanoma which transformed from primary acquired melanosis. During the surgery, Shields and team-applied cryotherapy and the use of topical mitomycin C. On other hand, Paridaens et al.^31^ reported 4 patients with conjunctival melanoma with one recurrence that was treated with mitomycin C. However, Espana et al. found no recurrences in the 30 month follow up 4 patients^33^. Similar to his results were the results by Dolla Pozza et al.^34^. In the study by Agraval et al. the authors reported numerous epibulbar melanomas of the conjunctiva excised with the covering the defect using AM. They did not mention relapses in the patients where histopathologically “in situ” melanoma was confirmed. In the group of 22 patients with invasive conjunctival melanoma the relapses were documented in five cases. In 2 of 5 patients, the need of total exenteration for orbital spread was necessary, the next 2 patients had in-transit metastases treated with proton beam therapy, but died from metastatic disease, and 1 patient in-transit metastases was treated locally with ruthenium plaque treatment and interferon injections. The recurrence did not occur at the site of the AM in all cases, they reported minimal complications, and the surgery led to good cosmetic results^25^.

For non-melanotic tumors, the safe and successful application of excision and AM use in covering the defects has been reported by several authors^27,33^. Palamar et al.^27^ treated 21 patients with ocular surface neoplasia (10 cases were invasive squamous cell carcinoma). They documented no cases of recurrence over a mean follow-up period of 30 months. The results of reconstruction have good cosmetic and functional results in most cases. Our results are very similar to those of Palamar et al.

Studies leading on the anti-cancer properties of AM are beginning to emerge in the literature. In our group, we documented minimal post-operative complications, for example, granuloma formation or scarring. In other studies, we found it interesting that the absence or very low recurrence of tumors appeared especially in the malignant cases. The “in vitro” studies, the authors suggested that the anti-cancer properties of AM are mediated by various mechanisms that include the induction of apoptosis and cell arrest. The mentioned molecular mechanisms are still under investigations^35–37^.

In the study by Agraval et al., they reported that the use of fresh AM during surgery has improved the local outcomes by improving healing and reducing scarring. They documented, a large, wider, complete excision of epibulbar lesions with non-touch technique reduces the risk of local recurrences. However, the large-conjunctival melanomas with in-transit conjunctival metastases have a not-good prognosis; but they support the use of fresh frozen AM in the management of suspicious conjunctival lesions requiring wide surgical excision^25^.

Wang et al. examined the tolerability and ocular comfort after surgery. All patients highlighted itching or moderate or mild ocular discomfort weeks after surgery, and mild or no discomfort one year later. This discomfort is in agreement has been described in the literature. The itching was probably associated with the sutures because all sutured patients showed moderate discomfort two weeks after surgery, whereas the fibrin glue ones reported none or mild discomfort^38^.

Regarding the cosmetic outcomes, Clearfield et al. reported the less encouraging results because three of our patients with AM (all males) had pterygium recurrence at 12 months follow-up, but without a clear association with age. Pterygium recurrence rate after AM implant has been described to be up to 42.3% at 6 months^39^. Other complications related to AM and fibrin glue have also been reported^40^, for example, graft dislocation, infection, bleeding, or pyogenic granuloma, but none of them were observed in our study.

Although pterygium excision with conjunctival autografting is currently considered the gold standard technique^38,41,42^, AM grafts are an effective alternative to conjunctival autografts because of the anti-inflammatory properties where the conjunctiva must be preserved^43^. However, in the epibulbar tumor surgery the defects can be more than two quadrants of the bulbar conjunctiva, and cosmetic outcomes can vary depending on the technique. Therefore, the AM covering of the defects is a crucial step of the surgical treatment.

## Conclusion

In comparison with our practical experience, AM grafts can be an effective alternative to defects covering after the removal of epibulbar lesions due to their anti-inflammatory properties. The main reason is that the conjunctiva must be preserved, and especially the most important, the application can be in malignant epibulbar conjunctival tumors.

The analysis of the investigated association between the number of quadrants infiltrated and malignancy showed that in our sample is clearly increasing trend, and this trend was found to be statistically significant. This result indicates that the association can be generalized.

## Data Availability

The datasets generated during and/or analyzed during the current study are available from the corresponding author on reasonable request.
